# Infectious Agents Induce Wnt/β-Catenin Pathway Deregulation in Primary Liver Cancers

**DOI:** 10.3390/microorganisms11071632

**Published:** 2023-06-22

**Authors:** Teresa Catalano, Federico Selvaggi, Diana Liberata Esposito, Roberto Cotellese, Gitana Maria Aceto

**Affiliations:** 1 Department of Clinical and Experimental Medicine, University of Messina, Via Consolare Valeria, 98125 Messina, Italy; 2 Unit of General Surgery, ASL2 Lanciano-Vasto-Chieti, Ospedale Clinicizzato SS Annunziata, 66100 Chieti, Italy; fedeselvaggi@hotmail.com; 3 Center for Advanced Studies and Technology (CAST), 66100 Chieti, Italy; diana.esposito@unich.it; 4 Department of Innovative Technologies in Medicine & Dentistry, “G. d’Annunzio” University of Chieti-Pescara, 66100 Chieti, Italy; 5 Department of Medical, Oral and Biotechnological Sciences, “G. d’Annunzio” University of Chieti-Pescara, Via dei Vestini 31, 66100 Chieti, Italy; roberto.cotellese@unich.it; 6 Villa Serena Foundation for Research, 65013 Città Sant’Angelo, Italy

**Keywords:** primary liver cancer, Wnt/β-catenin, gut–liver axis, microorganisms, microbiota, HCC, CCA, infections, HBV, HCV

## Abstract

Interaction between infectious agents and liver tissue, as well as repeated and extreme biological events beyond adaptive capacities, may result in pathological conditions predisposing people to development of primary liver cancers (PLCs). In adults, PLCs mainly comprise hepatocellular carcinoma (HCC) and cholangiocarcinoma (CCA). Various infectious agents in the hepatic microenvironment can destabilize normal liver cell functions by modulating the Wnt/β-catenin pathway components. Among them, hepatotropic viruses B, C, and D are involved in Wnt/β-catenin signaling dysregulation. Other microbial agents, including oncogenic viruses such as Epstein–Barr virus (EBV) and human papilloma virus (HPV), bacteria, e.g., *Mycoplasma hyorhinis* and *Salmonella Typhi*, the protozoan parasite *Toxoplasma gondii*, the fungus *Aspergillus flavus*, and liver flukes such as *Clonorchissinensis* or *Opisthorchis viverrini*, may induce malignant transformation in hepatocytes or in target cells of the biliary tract through aberrant Wnt signaling activation. This review focuses on new insights into infectious agents implicated in the deregulation of Wnt signaling and PLC development. Since the Wnt/β-catenin pathway is a driver of cancer following viral and bacterial infections, molecules inhibiting the complex axis of Wnt signaling could represent novel therapeutic approaches in PLC treatment.

## 1. Introduction

According to global cancer 2020 estimates, primary liver cancer (PLC) is the sixth most common cancer worldwide and represents the third cause of tumor death [1]. In adults, PLCs include hepatocellular carcinoma (HCC) and cholangiocarcinoma (CCA) in proportions of 90% and 5–10%, respectively [2]. HCC is the third most common cause of cancer-related deaths worldwide, and CCA is the second most prevalent type of liver cancer [3,4]. Despite efforts in basic and clinical research, the five-year survival rate of patients affected with PLCs is less than 5%, since diagnosis is often delayed and is associated with advanced stage [5]. Therefore, it is of fundamental importance to reduce incidence and impact of these diseases through knowledge of risk factors and primary prevention strategies. 

Different risk factors are involved in HCC and CCA pathogenesis and are related to diet, environmental exposure, lifestyle, and genetic predisposition [6,7,8]. Indeed, the interaction of liver tissue with infectious agents through the course of life or the detrimental impact of frequent and extreme biological events exceeding adaptive capacity, may determine numerous disorders predisposing humans to the onset of PLC [6,9,10]. Pathological liver conditions that predispose humans to PLCs include metabolic dysfunction-associated fatty liver disease (MAFLD), chronic hepatitis, alcoholic cirrhosis and non-alcoholic fatty liver disease (NAFLD) [9]. 

Patients affected with NAFLD show a higher risk of developing bacterial, fungal, and viral infections, but the exact mechanism by which this occurs is still unknown [11]. Hepatitis B virus (HBV) infection could stimulate NAFLD progression to liver steatosis, through the induction of cholesterol synthesis gene expression [12]. Even though the prevalence of HCC from NAFLD is less common than that from chronic viral hepatitis B and C, hepatocellular carcinoma usually arises in the setting of chronic liver damage and cirrhosis [10]. Indeed, both hepatitis C virus (HCV) and HBV are associated with HCC development, whereas HCC related to other underlying hepatic diseases is linked to the onset of cirrhosis [6]. 

Multiple risk factors have been linked to the development of CCA. These include hepatic parasites, chronic biliary tract, and liver diseases, including primary sclerosing cholangitis, choledochal cysts, Caroli’s disease and HCV cirrhosis, as well as lifestyle factors associated with chronic hepatic inflammation and cholestasis [13,14,15,16]. CCA occurs along the biliary tree and is classified into intrahepatic, perihilar, or distal [17]. The prevalence of CCA varies in different geographical areas, because of environmental and genetic conditions. This disease is observed after the fourth decade of life and more often in men [16]. 

In recent decades, a clear causal link has been found between liver damage, inflammation and regeneration and the occurrence of PLCs. Chronic inflammation determined by long-term infections is observed in more than 80% of the HCCs and represents an important factor able to modify the hepatic microenvironment [2]. The liver, which receives portal and systemic circulation, contains about one third of the reticuloendothelial system and acts as a defense against infection [18]. This allows the liver to maintain the equilibrium between host immune system activation and tolerance to circumvent inappropriate immune responses against non-pathogenic exogenous molecules, such as food-antigens [19]. The involvement of the gut–liver axis in the pathophysiological mechanism responsible for the development of HCC has recently been revealed [20,21,22]. The gut barrier integrity protects the host from the microbiota residing in the intestinal ecosystem. If this barrier is leaky or disrupted and/or in the presence of gut dysbiosis, the liver is exposed to factors from the intestine, such as bacteria and bacterial endotoxins (e.g., LPS) that can lead to hepatic diseases [23,24]. Increased bacterial translocation from the gut could be a key factor associated with HCC development in animal models, although data coming from human studies are still incomplete [22]. Moreover, depletion of the gut microbiota in mice could reduce T cell antiviral immunity in the liver, and protract HBV infection [25]. Various microorganisms can exert different clinical effects on the liver, such as increasing levels of aminotransaminases and causing acute liver failure and hepatic fibrosis, rapidly progressing to cirrhosis [19,26]. Significant modifications occur in tissue metabolism throughout inflammatory and immune responses. They consist of local reduction of nutrients, increased oxygen consumption, and enhanced generation of reactive oxygen and nitrogen intermediates. In addition, evolutionary adaptations allow pathogens to utilize host metabolic pathways and survival mechanisms [27]. 

**Figure 1:**
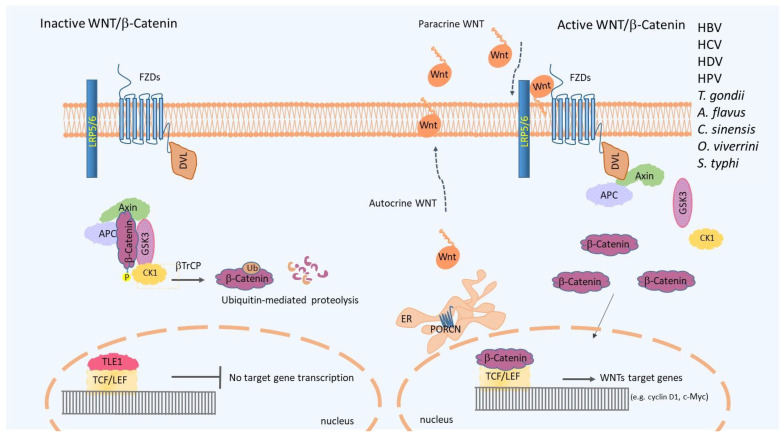
Overview of the Wnt/β-catenin signal pathway. Wnt ligands o-acylated by Porcupine acylase (PORCN) can be secreted and exert both paracrine and autocrine actions on cells. In the absence of adequate ligand production, β-catenin is initiated for proteasomal degradation by the destruction complex (Axin, APC, GSK-3β), sequentially phosphorylated by CK1 and ubiquitinated by β-TrCP. In the nucleus, in the absence of β-catenin, transcription of target genes is repressed by the binding of TLE-1 to the transcription factor TCF/LEF. In the presence of Wnt ligand overproduction, Frizzled and LRP coreceptors associate by recruiting Axin and Dvl to the membrane; the inactive signal then becomes active, the stabilized β-Catenin can enter the nucleus, through binding with the transcription factor TCF/LEF, and can induce the expression of Wnt target genes (e.g., cyclin D1 or c-Myc). The main infectious agents that have been shown to interfere with the Wnt/β-catenin pathway are: *Hepatitis B virus* (*HBV*); *Hepatitis C virus* (*HCV*); *Hepatitis D virus* (*HDV*); *Human papilloma virus* (*HPV*); *T. (Toxoplasma) gondii*; *A. (Aspergillus) flavus*; *C. (Clonorchis) sinensis*; *O. (Opisthorchis) viverrini*; *S. (Salmonella) typhi*.

The Wingless/It (Wnt)/β-catenin signaling pathway is a pathway preserved throughout animal evolution. It is involved in the regulation of cells’ differentiative fates both during embryonic development and in the homeostasis of tissue regeneration in the adult organism [28,29]. Given its involvement in both stem capacity and cell differentiation, alterations that overregulate the functions of the Wnt signaling are often observed in tumors, particularly in those tissues that physiologically depend on it for their ability to renew or repair themselves [30]. The signal is activated by a family of Wnt ligands consisting of 19 secreted glycoproteins that can act in both autocrine and paracrine manners, binding to frizzled (Fzd) receptor complexes on the cell membrane to transduce downstream intracellular signals [31]. At the cytoplasmic level, the signal network is defined according to whether it is dependent on its key mediator, β-catenin; thus, the pathway is divided into canonical (β-catenin-dependent) or non-canonical (β-catenin-independent) [32]. β-catenin, as a scaffold protein, can link the cytoplasmic tail of E-cadherin to the actin of the cytoskeleton [33]. In the presence of receptor stimulation by Wnt ligands, cytoplasmic and nuclear levels of β-catenin increase due to the reduction of its proteasomal degradation. The process is controlled by a multi-protein destruction complex consisting of Axin, Adenomatous polyposis coli (APC), Casein kinase1a (CK1a) and Glycogen synthase kinase 3-β (GSK3-β) [34] (Figure 1). The continuous elimination of β-catenin prevents it from reaching the nucleus and the Wnt target genes are then repressed by the DNA-bound T cell factor/lymphoid enhancer factor (TCF/LEF) proteins [35] (Figure 1).

Wnt signaling plays key physiological functions in the liver, from development to metabolic zonation and regeneration after damage [36,37]. Specifically, β-catenin and its negative regulator APC are localized in the perivenous and periportal areas, where they control liver metabolic activity [38]. Molecular dysregulation of the Wnt pathway is strongly associated with the hepatic microenvironment and may drive the progression from precancerous dysplasia to PLC metastasis [26,39,40,41]. This is a common condition also reported in liver metastasis from colorectal cancer [42]. 

Upregulation of Wnt ligands and their receptors/coreceptors, as well as downregulation of Wnt/β-catenin signaling inhibitors, have been reported in liver cancer and its adjacent precancerous lesions [43]. Hepatic tumors recurrently show mutations in genes encoding key elements of the Wnt/β-catenin pathway [26,39,40]. Mutations of the *AXIN* and *APC* genes, and the *CTNNB1* gene encoding β-catenin, have been detected in PLCs [41,44,45,46]. 

Infectious agents implicated in PLC development can modulate Wnt pathway molecular components to destabilize normal cell functions [47]. In fact, oncogenic viruses and bacteria toxins often activate an aberrant Wnt signaling that promotes malignant transformation in target cells [24,47]. 

A better understanding of the events that lead to dysregulation of the Wnt signal following infection by microbial agents in PLCs could lead to a targeted therapeutic approach in liver cancer treatment. Therefore, it is important to identify the infectious agents that alter the Wnt pathway in liver cells, with the aim to understand the mechanisms used by bacteria or viruses or parasites to modify the Wnt signal and evaluate their involvement in the pathogenesis of HCC and CCA. This review considers what is currently known about deregulation of Wnt signaling due to the presence of infectious agents in the hepatic microenvironment as a driver of liver carcinogenesis. Comprehension of the cellular and molecular events occurring in the microenvironment in the presence of infectious agents could improve targeted therapeutic approaches in the treatment of PLCs. 

## 2. Infectious Agents Involved in HCC

### 2.1. Hepatitis B Virus (HBV)

In approximately 80% of HCCs, the development of disease is associated with HBV and/or HCV chronic infections [48]. Without any doubt, the vaccination and therapeutic treatment of hepatitis B, in addition to programs to prevent HBV and HCV transmission, are currently changing the epidemiology of HCC, although its non-viral etiology is increasing [49,50,51]. HBV seroprevalence shows the highest incidence rate in Africa and Western Pacific Asia, while chronic HBV disease is decreasing in many European countries; males over 40 years of age are most affected worldwide [52,53]. From a molecular point of view, Wnt is one of the main pathways modulated by HBV [54] (Table 1). Indeed, regulatory hepatitis B Viral X protein (HBx) and hepatitis B surface antigen (HBsAg) can activate Wnt/β-catenin signaling components [55]. In particular, HBx protein regulates the expression of the secreted frizzled-related proteins, SFRP1 and SFRP5, which antagonize Wnt This occurs through hypermethylation of their gene promoter by the DNA methyltransferases DNMT1 and DNMT3 [56]. In addition, HBx impairs the cell destruction complex through multiple mechanisms [41], such as the competitive binding to the β-catenin domain of APC, or the Src kinase activation that suppresses GSK-3β, or the induction of the androgen receptor pathway by cell-cycle-related kinase [41,57]. Furthermore, HBx increases the expression of ETS variant 4 (ETV4) through Disheveled 2 (DVL2) transcriptional activation and consequent inhibition of β-catenin degradation; this Wnt deregulation is associated with HCC progression and poor prognosis [58]. ETV4 also occurs in Wnt/β-catenin pathway activation through the upregulation of Annexin A2 (ANXA2) in HBV-liver cancers [59]. On the other hand, HBsAg upregulates the expression of LEF-1 transcription factor in HCC cell models and tissues [60,61]. A recent study shows that the intermediate protein p22, a precursor to HBVe antigen (HBeAg), can activate TCF/β-catenin transcription in in vitro and in vivo models [62]. Regarding the role played by gene mutations, changes in Hbx modulate the expression of Wnt-5a in hepatoma cells, whereas inactivating mutations in *APC*, *AXIN1* and *AXIN2* genes or methylation in *APC* aberrantly modify the Wnt signaling response [63,64]. In HBV-dependent HCC, *AXIN1* mutations are less frequently associated with *CTNNB1* ones than those found in HCV-related HCC [39,65]. Polymorphisms in *AXIN1*, *AXIN2*, *CTNNB1*, and *WNT2* genes seem to confer a different HBV-dependent HCC susceptibility and progression [66]. In addition, *WNT2* and *WNT1* gene expression can be considered as diagnostic or prognostic biomarkers, respectively, while Wnt-3a protein expression could be a novel HCC target in early diagnosis [67,68]. Activation of the Wnt/β-catenin pathway could also be induced by integration of the HBV genome into long interspersed nuclear element 1 (LINE1) with production of an oncogenic chimeric transcript, although this hypothesis is not fully confirmed [64,69]. HBV can also indirectly influence the Wnt signaling in hepatocarcinogenesis through the modulation of the expression of different miRNAs, such as miR-26a, miR-15a, miR-16-1, miR-148a, miR-132, miR-122, miR-34a, miR-21, miR-29a, miR-222 and miR-119a/b-3p [70]. In HBV-related HCCs and matched adjacent non-cancerous liver tissues, a differential regulation of the Wnt-β-catenin pathway, but no variability of protein levels or phosphorylation status, was identified by integrated proteogenomic profiling [71]. Recently, mRNAs of some Wnt target genes in HBV-associated/*CTNNB1*-mutant and *TP53*-mutant HCCs were found to be differentially expressed and associated with the loss of epithelial phenotype. This condition most likely resulted from altered phosphorylation of proteins implicated in the actin filament organization or decreased expression of the epithelial markers E-cadherin (CDH1) and Keratin-19 (KRT19). *CTNNB1*-mutant HCCs are often low grade and linked with the immune-desert phenotype, while *TP53*-mutant HCCs are related to HBV infection [72]. In addition, the presence of HBV can affect normal liver cell processes when it crosses a developmental pathway, such as Wnt/β-catenin [26,47].

### 2.2. Hepatitis C Virus (HCV)

Chronic HCV infection affects an estimated 115 million people worldwide; this disease is more common in East/Central Asia and North Africa than in Europe [53]. 

HCV core protein, non-structural protein 4B (NS4B) and 5A (NS5A) are able to activate Wnt/β-catenin signaling in HCC pathogenesis (Table 1). The core protein upregulates Wnt ligands, Fzd and LRP5/6 receptors, increases the TCF-dependent transcription stimulated by Wnt3a and downregulates Wnt antagonists Dickkopf (DKK), SFRP1 and E-cadherin [73,74,75,76]. HCV core protein can reduce E-cadherin expression levels due to *CDH1* gene promoter hypermethylation, with consequent dissociation of the β-catenin/E-cadherin complex, and stimulates tumor cell growth by GSK-3β inactivation and Wnt3a release [76,77]. A study performed on core proteins of HCV subtypes from Cambodia, Romania and Cameroon detected a critical role of the core region encompassing amino acids Ser64 and Thr71 in the upregulation of Wnt target genes *c-MYC* and Cyclin D1 (*CCND1*), and different nuclear levels of β-catenin in cells infected, according to various HCV subtypes [78].

NS4B activates the Wnt3a-induced Wnt/β-catenin signaling in HCC Huh7 cells and normal human liver LO2 cell lines [77,79]. NS5A stabilizes β-catenin and stimulates its transcriptional activity with a mechanism mediated by phosphoinositide 3-kinase (PI3K)-AKT pathway activation and subsequent inactivation of GSK-3β [80,81,82]. 

The progression from chronic HCV hepatitis to HCC is favored by binding of Wnt1 and Wnt5a to Fzd, which transactivates the EGFR pathway [83]. In a model of HCV infection in vitro, activation of Wnt/β-catenin signaling remains even after antiviral treatment and is related to GSK-3β inhibition [84]. In HCV-related HCC, FGF signaling induces the release of phosphorylated β-catenin from the complexes with E cadherin [83]. 

Downregulation of APC or Axin2 induced by HCV increases de-phospho-β-catenin, which translocates to the nucleus to activate the target genes and induce malignant changes in hepatocytes [85]. Chronic HBV and HCV hepatitis shows increased activity of cyclooxygenase 2 (COX2) that in turn activates the Wnt pathway through the release of prostaglandin E2 [86]. HCV also downregulates APC and Axin2, while it upregulates miR-155, which promotes Wnt signaling activation [87]. Conversely, miR-125b correlates with APC through suppression of cell growth, arrest of cell cycle at G1 phase, and inhibition of HCC cell proliferation, migration and invasion [88].

### 2.3. Hepatitis D Virus (HDV)

HDV is a defective RNA virus that needs co-infection with HBV. At a global level, approximately 5% (15–20 million) of chronic HBV carriers are co-infected with HDV. HDV infection may occur with HBV co-infection or superinfection in persistent HBV hepatitis [89]. HDV produces the small and large hepatitis delta antigens (S-HDAg and L-HDAg, respectively). Nuclei of Huh-7 cells transfected with the L-HDAg expression plasmid show β-catenin immunoreactivity. L-HDAg activates the transforming growth factor-β (TGF-β), which may induce epithelial–mesenchymal transition (EMT) and fibrosis [90].

### 2.4. Epstein–Barr Virus (EBV)

EBV has a high global prevalence and is present in more than 90% of adults [91]. In HCV-related HCC, EBV coinfection may promote carcinogenesis through increased HCV replication and inflammation [92,93,94]. Research has not confirmed the direct involvement of EBV in liver carcinogenesis [95,96,97]. A more recent study detected the frequent activation of EBV in infiltrating lymphocytes of clinically low aggressive HCCs [94]. Based on results coming from other cancers, EBV might induce epigenetic reprogramming of LEF1 and Wnt5a. In other terms, EBV might indirectly contribute to hepatocarcinogenesis [98,99]. In light of this preliminary evidence, it might be interesting to investigate the role of EBV in the dysregulation of Wnt signaling in HCC. Viral interference, which can inhibit both GSK-3 and promote β-catenin functions, has been observed with DNA virus-encoded proteins, such as the X protein of HBV and the EBV LMP2A protein [100]. 

### 2.5. Human Papilloma Virus (HPV)

Prevalence and distribution of over 207 HPV genotypes vary according to different populations and geographical areas [101]. Some reports have investigated the role of HPV-16 in hepatocarcinogenesis by detecting the common involvement of E6 oncoprotein and HBx in AP-1 activation to increase HBV transcriptional activity in human liver cells [102]. E6 and E7 oncoproteins are involved in the regulation of the Wnt/β-catenin pathway, which results in hyperactivated HPV-associated cancers [103,104]. There is currently no further experimental evidence on the role of HPV in liver cancer.

### 2.6. Non-Viral Infectious Agents Involved in HCC

Regarding the role of non-viral agents in liver carcinogenesis, it has been reported that *Toxoplasma gondii*, a protozoan parasite, is involved in liver disease and fibrosis, with a global distribution. Indeed, in China, the highest seroprevalence of *T. gondii* was found among patients with liver disease, who ranged in age between 41–50 years [105]. *T. gondii* secretes GRA18, a protein exported to the cytoplasm of infected cells, in which it is a potential inhibitor of the β-catenin destruction complex [106]. In the presence of GRA18, β-catenin moves towards the nucleus where it induces the expression of anti-inflammatory chemokines Ccl17, Ccl22, and Ccl24 [106]. *Echinococcus granulosus* seems to significantly extend the survival and play a protective effect in HCC patients [107]. The presence of *Mycoplasma hyorhinis*, which predominantly infects swine, has been also detected in human cancer tissues [108]. *M. hyorhinis* infection promotes HCC progression and cell migration. The interaction between mycoplasma p37 membrane protein and the epithelial cell adhesion molecule (EpCAM) involves the Akt/mTOR pathway [109], which in turn can control Wnt/β-catenin signaling. Since in gastric cancer *M. hyorhinis* infection has been reported to induce nuclear β-catenin accumulation and increase its target gene expression [110], further research is needed to assess the direct involvement of *M. hyorhinis* in hepatocarcinogenesis, by Wnt signaling dysregulation. 

In some countries, such as India, Mexico, Pakistan, Sudan, and Saudi Arabia, *Aspergillus flavus* has a high prevalence and is frequently isolated in the respiratory tract [111]. Aflatoxin-B1 (AFB1) mycotoxin, produced by *Aspergillus fungi* (*flavus*), is a genotoxic compound responsible for HCC. Persistent AFB1 exposure activates the Wnt pathway [112]. Other studies have revealed the role of AFB1 in miR-33 and miR-34a activation to downregulate the Wnt/β-catenin pathway in HCC cells, suggesting an involvement for these miRNAs in therapeutic applications [113,114]. Moreover, macrophages infected with *A. flavus, A. fumigatus*, and *Candida albicans* strongly activate Wnt signaling [115]. These contradictory data indicate that probably other unknown factors are involved in the modulation of Wnt signaling by mycotoxins produced by *A. flavus*. Finally, AFB1 may increase the HCC risk in the presence of HBV or HCV infections [116,117]. 

## 3. Infectious Agents Involved in CCA 

The onset of CCA is related to a chronic inflammatory condition due to long exposure of the cholangiocytes to predisposing risk factors, such as transmissible agents (Table 1). In bile ducts, this persistent phlogistic state is responsible for the induction of several pathways, including Wnt signaling, as well as the activation of cell proliferation and CCA-predisposing genetic mutations and epigenetic changes [118]. 

In this tumor, mutations in the *CTNNB1* (1.5%), *AXIN1* (4%) and *APC* (2%) genes are the most commonly identified [41]. The host response to infection causes differential expression of Wnt signaling molecules, in particular overexpression of canonical ligands such as Wnt7b and non-canonical ligands such as Wnt5a, in infiltrating macrophages activated in both inflammation and cancer [99,119]. Moreover, macrophages determine the secretion of pro-inflammatory cytokines activating Wnt5a [120]. Tumor-associated macrophages (TAMs) are implicated in CCA cell proliferation, metastasis, and angiogenesis as well as in the activation of Wnt/β-catenin signaling [121]. In fact, this pathway contributes to CCA induction, progression, EMT and multidrug resistance [26]. Other signaling pathways, such as PI3K/AKT/PTEN/GSK-3β, in association with microRNA upregulation, are involved [122]. 

### 3.1. Hepatitis B Virus (HBV)

HBV genes are present in the genome of CCA. HBx protein may be implicated in cholangiocarcinogenesis, since HBx gene insertion induces cis-activation of Telomerase reverse transcriptase (*TERT*) mRNA transcription in CCA cell lines [123]. An aberrant activation of Wnt/β-catenin signaling was observed in carcinogen-treated HBx transgenic mice, which developed mixed HCC and CCA in the same liver [124]. 

### 3.2. Hepatitis C Virus (HCV)

Differing from HBV-associated CCA, HCV infection is associated with a poor prognosis of this type of cancer [83,125]. Molecular mechanisms inducing CCA after HCV infection are still unclear [126]. To the best of our knowledge, there is not enough scientific evidence regarding the involvement of the Wnt pathway in the pathogenesis of HCV-induced CCA, and there is a lack of studies on a broader case series. Analysis of few samples of CCA and hepatocholangiocarcinoma showed no mutations in *TERT* promoter and *CTNNB1* exon 3 [127]. 

### 3.3. Human Polyomavirus (HPyV)

In CCA lesions and adjacent peritumoral cells of bile duct epithelium, the occurrence of *human polyomavirus 7* (*HPyV7*) is more frequent than *human polyomavirus 6* (*HPyV6*) and oncogenic *Merkel cell polyomavirus* (*MCPyV*) [128]. 

### 3.4. Epstein–Barr Virus (EBV)

EBV can synergistically cooperate with HBV in the onset of a rare subtype of intrahepatic CCA (EBVaICC), a tumoral form distinguished from others by exclusive clinicopathological and genetic alterations [129] and probably with a different pathogenesis that does not seem to involve Wnt signals. 

### 3.5. Non-Viral Infectious Agents Involved in CCA

Mechanisms leading to CCA development are dependent on genetic alterations induced by different trematodes. Liver flukes such as *Clonorchissinensis*, *Opisthorchis viverrini* and *Opisthorchis felineus* are trematodes of the family Opisthorchiidae. In particular, the genera *Clonorchis* and *Opisthorchis* are responsible for CCA [130,131].

*C. sinensis* determines clonorchiasis, which is correlated with hyperplasia of biliary epithelium and metaplasia of mucin-secreting cells in bile ducts, probably through upregulation of Wnt7b, Fzd6 and cell cycle controllers, which leads to CCA development [132]. An excretory/secretory (ES) product of *C. sinensis*, the growth factor-like protein termed granulin, seems to be involved in cell migration and invasion in CCA as well as in HCC, with activation of EMT by upregulation of β-catenin and decline of E-cadherin [133]. To support this, in a mouse model of *C. sinensis* infection, macrophages showed variations in polarization and phenotype during different phases of disease [121]. On the other hand, ES products of *O. viverrini* contain growth factors and other molecules, including liver fluke granulin. In Opisthorchiasis, chronic inflammation is induced by mechanical damage to biliary epithelia caused by *O. viverrini*, which continuously exposes cholangiocytes to granulin and other molecules [134]. The chronic inflammation may act through receptor tyrosin kinase (RTK) signaling, which is related to MAPK, PI3K/AKT and Wnt/β-catenin pathways [135]. 

In particular, the development of opisthorchiasis-dependent CCA is associated with upregulation of the Wnt/β-catenin signaling pathway. In cholangiocarcinogenesis of *O. viverrini*-infected hamsters, increased expression of Wnt3, Wnt3a, Wnt5a, Wnt7b as well as β-catenin nuclear translocation were found [136]. Similarly, these molecules were also upregulated in human CCA tissues. On the other hand, some Wnt ligands (i.e., Wnt3a) released into the tumor microenvironment could be produced by both CCA and inflammatory cells [137]. 

*Cryptosporidium parvum* has been isolated in human immunodeficiency virus (HIV) cholangiopathy. *C. parvum* induces a Toll-like receptor 4-dependent response that is inhibited by HIV-1 trans-activator of transcription (Tat) protein [138]. Possible retrograde invasion of *C. parvum* from the small intestine to biliary ducts favors a pro-inflammatory cholangiopathy that involves molecules such as micro-RNA let-7i, NF-kB p50-CCAAT/enhancer-binding protein beta (C/EBPbeta) and NRAS [139]. Moreover, HIV-1 Tat increases apoptosis in cholangiocytes induced by *C. parvum* through activation of the Fas/Fas ligand pathway [140]. In individuals with CD40L deficiency, *C. parvum* infection predisposes humans to sclerosing cholangitis, which may evolve to CCA [141]. Wnt/β-catenin signaling interferes with maturation and activation of dendritic cells in the tumor microenvironment. Inflammatory dendritic cells express elevated levels of costimulatory molecules, including CD40 [142]. 

*Helicobacter pylori* was found to be associated with opisthorchiasis and hepatobiliary disease [143]. It induces the Wnt/β-catenin pathway and macrophage recruitment [144].

*Salmonella typhi*. Chronic carriers for *S. typhi* show a 200-fold risk of developing CCA [131]. In intestinal stem cells of a murine model, *Salmonella enterica* serovar Typhimurium increases mRNA expression of Wnt2, Wnt3, Wnt6, Wnt9a, Wnt11 as well as Fzd2, Fzd4, Fzd6, Fzd7, Fzd8, and Fzd9 [145,146,147]. *S. enterica* serovar Typhimurium could dysregulate the Wnt/β-catenin signaling by the translocation of antigens through the gut–vascular barrier, with a mechanism based on its pathogenicity island (Spi) 2–encoded type III secretion system that permits its dissemination in the liver and bloodstream [148]. Induction of Wnt2 and Wnt11 expression has been partially ascribed to Salmonella AvrA, which is a bacterial effector involved in the control of β-catenin ubiquitination and stabilization [99]. These data suggest a deep role of *Salmonella* in the activation of the Wnt pathway in CCA.

**Table 1:** Summary of infectious agents interfering with Wnt/β-catenin signalling in hepatocellular carcinoma (HCC) and cholangiocarcinoma (CCA).

Diseases and *Pathogens*	Mode of Entry into the Liver	Wnt Pathway Deregulation	Determinants	References
**HCC**				
*Hepatitis B Virus*	bloodstream	SFRP1 and SFRP5 downregulation. Competitive binding to β-catenin domain of APC; GSK3-β suppression. LEF-1 upregulation. Activation of TCF/β-catenin transcription. Inactivating mutations in *APC*, *AXIN1* and *AXIN2*. *APC* methylation. Polymorphisms in *AXIN1*, *AXIN2*, *CTNNB1*, and *WNT2*.	HBx HBsAg p22	[41,54,57,60,61,62,63,64]
*Hepatitis C Virus*	bloodstream	Wnt, Fzd and LRP5/6 overexpression; increased TCF-dependent transcription; DKK and SFRP1 downregulation. *CDH1* hypermethylation. Wnt3a induction. Stabilization of β-catenin by GSK-3β inactivation. β-catenin-EGFR pathway transactivation by Wnt1 and Wnt5a. APC or Axin2 downregulation. Wnt signaling activation by miR-155 upregulation.	HCV core protein NS4B NS5A	[73,74,75,76,77,79,81,82,83,85,87,90]
*Hepatitis D virus*	bloodstream	β-catenin increased expression	L-HDAg	[90]
*Human papilloma virus*	possibly bloodstream	Wnt/β-catenin pathway hyperactivation	E6 E7	[103,104]
*Toxoplasma gondii*	mesenteric and portal vessels	β-catenin destruction complex inhibition	GRA18	[106]
*Aspergillus flavus*	mesenteric and portal vessels	Wnt pathway activation. Wnt/β-catenin signaling downregulation by AFB1 activated by miR-33a and miR-34a	AFB1	[112,113,114]
**CCA**				
*Hepatitis B Virus*		Aberrant activation of Wnt/β-catenin signaling	HBx	[124]
*Hepatitis C Virus*		Unknown		
*Human polyomavirus 6* *Human polyomavirus 7* *Merkel cell polyomavirus*		Unknown		
*Epstein-Barr virus*		Unknown		
*Clonorchis sinensis*	retrograde invasion from the small intestine to biliary ducts	Wnt7b and Fzd6 overexpression. β-catenin upregulation	Granulin	[132,133]
*Opisthorchis viverrini*	retrograde invasion from the small intestine to biliary ducts	Wnt3, Wnt3a, Wnt5a, Wnt7b, and β-catenin overexpression	Granulin	[136]
*Cryptosporidium parvum*	retrograde invasion from the small intestine to biliary ducts	Wnt/β-catenin signaling affects dendritic cells maturation and activation in tumor microenvironment	Unknown	[142]
*Salmonella Typhi*	hematogenous seeding in the liver during bacteremic phase, and reticuloendothelial cells infection	Wnt2, Wnt3, Wnt6, Wnt9a, Wnt11 and Fzd2, Fzd4, Fzd6, Fzd7, Fzd8, Fzd9 mRNA upregulation. Wnt2 and Wnt11 expression induction	AvrA	[99,145,146,147]

Hepatitis B Viral X protein (HBx); hepatitis B surface antigen (HBsAg); *Hepatitis C Virus* (HCV) core protein; non-structural protein 4B (NS4B); non-structural protein 5A (NS5A); large hepatitis delta antigen (L-HDAg); E6 oncoprotein; E7 oncoprotein; dense granule (GRA) protein (GRA18); aflatoxin-B1 (AFB1); Salmonella bacterial type III secretion effector protein AvrA.

## 4. Therapeutic Perspectives

Kinases involved in the Wnt signaling pathway could represent attractive molecular targets for their inhibitors and antiviral agents in the treatment of infectious agents responsible for liver cancer. A novel therapeutic target in HCV-infected patients could be GSK-3, which constitutively phosphorylates cytosolic β-catenin. GSK-3 is expressed as GSK-3α and GSK-3β isozymes, which are structurally similar in catalytic domains [149]. Under physiological conditions, GSK-3 activity is inhibited through phosphorylation of its serine residues by several kinases, such as protein kinase A (PKA), protein kinase C, Akt. These changes lead to Wnt cascade attenuation and maintenance of cellular homeostasis. Conversely, overexpression of GSK-3 or imbalance of its phosphorylation status induce overactivity of the enzyme [149,150]. HCV infection increases cyclic adenosine monophosphate (cAMP) levels necessary for PKA activation. Therefore, the use of endoplasmic reticulum (ER) stress/PKA/GSK-3β-dependent Wnt/β-catenin signaling as targets of PKA inhibitor could constitute a new therapeutic approach for patients infected with HCV, in which the Wnt/β-catenin pathway is activated despite treatment with direct-acting antiviral agents (DAAs) and HCV clearance [150]. Another therapeutic strategy could be based on the reduction of GSK3β in tumor-associated macrophages (TAMs) to prevent HCC development through the inhibition of M2 phenotype and increase the sensitivity of anti-programmed death 1 (PD1) immunotherapy by the decrease of Programmed Death-Ligand 1 (PD-L1) ubiquitination [151]. 

Other interesting therapeutic perspective is represented by targeting of the β-catenin destruction complex (APC, Axin, CK1, and GSK3β) with the aim to induce the inactivation of Wnt signaling in HCC. The protein destruction complex induces degradation through CK1- and GSK3-mediated phosphorylation of β-catenin, followed by enrollment of the beta-transducin repeats-containing protein (β-TrCP) ligase [43]. Targeting the intracellular protein complex is difficult since APC mutations are often present. Stabilization of the β-catenin protein destruction complex allows inactivation of the Wnt pathway. Tankyrase (TNKS) is an activator of the Wnt/β-catenin cascade since it degrades the negative regulators of β-catenin, AXIN1 and AXIN2, through proteasomal degradation mediated by ubiquitin [43,152]. TNKS inhibitors negatively regulate Wnt signaling by stabilizing AXIN and antagonizing the Wnt/β-catenin pathway. Among them, XAV939 and WXL-8 reduce Wnt/β-catenin cascade and inhibit HCC cell proliferation. NVP-TNKS656 inhibits HCC cell line proliferation, as well as invasion, metastasis, and EMT features [153]. Indeed, inhibitors of TNKS could represent a novel attractive therapeutic target in liver cancers induced by infective microorganisms. Additional molecules act on the protein destruction complex at different levels. Pyrvinium blocks Wnt signaling through the increase of CK1 kinase activity, whereas the non-steroidal anti-inflammatory drug Sulindac binds to Dvl [154]. Currently, these therapies are still in the experimental stage, and it is hoped that they can be used in the treatment of primary liver cancers with infectious etiology. Deubiquitinase inhibitors may represent an alternative therapeutic option to reduce β-catenin levels by promoting its degradation [155]. In their evolution, pathogens have developed molecular signals that enable them to survive host defenses. Recent studies have unveiled the role of the ubiquitin system in innate immunity pathways in controlling responses to infectious agents. The ubiquitination process is involved in post-translational regulation of intracellular proteins through the control of their degradation via the proteasome system [156,157]. Deubiquitinating enzymes (DUB) are a large group of proteases implicated in deubiquitylation, which can rescue labelled substrate proteins by removing their conjugated ubiquitin chains [158]. Recent findings have revealed the role of viral-derived DUBs in counteracting host immune responses [159] and DUBs have also been proposed as possible therapeutic targets for the treatment of infectious diseases [160,161]. Viral-derived DUBs are not structurally homologous to those of the host, but they can act on the same target proteins [162,163]. In the case of viruses, the production of DUBs prevents them from being eliminated by host cells. 

The identification of microbial DUBs, capable of interfering with the molecular pathways of the host cell, could be important for the development of inhibitory drugs. Therefore, it would be interesting to understand whether infectious agents, related to the occurrence of primary liver tumors, produce DUBs that inhibit the proteasomal degradation of β-catenin. 

## 5. Conclusions

Pathogens alter Wnt-associated processes to increase both infection and survival in the human host. In conserved eukaryotes, Wnt signaling is involved in the interplay between the human host and extracellular and intracellular bacterial pathogens [164]. These aspects have important implications in the knowledge of microbic carcinogenesis, especially in the human liver. Since the Wnt/β-catenin pathway is considered a driver of viral and bacterial tumors, the development of targeted molecules that are able to inhibit this intracellular signaling might improve the therapeutic approaches of patients with PLCs. In recent years, studies have focused on the mechanisms used by GSK-3 and β-catenin to control the antiviral innate immune response toward RNA and DNA virus infections. Targeting the GSK-3/β-catenin axis could avoid harmful effects consequent to chronic infection, such as viral oncogenesis. Nevertheless, detection of small molecules inhibiting the interaction of β-catenin with TCF and TNKS has not given the expected results [100]. DUB inhibitors that reduce the levels of β-catenin by promoting its degradation [156] represent an alternative therapeutic option.

A better insight into the molecular mechanisms leading to dysregulation of Wnt signaling during infections could open up new perspectives in clinical practice with integrated therapies in PLC patients.

## Data Availability

Not applicable.
